# The role of chaperones in iron–sulfur cluster biogenesis

**DOI:** 10.1002/1873-3468.13245

**Published:** 2018-10-01

**Authors:** Rita Puglisi, Annalisa Pastore

**Affiliations:** ^1^ UK Dementia Research Institute at King's College London London UK; ^2^ The Wohl Institute at King's College London London UK; ^3^ Department of Molecular Medicine University of Pavia Pavia Italy

**Keywords:** Chaperones, iron–sulfur cluster, neurodegeneration, protein folding, structural biology

## Abstract

Iron–sulfur cluster biogenesis is a complex process mediated by numerous proteins among which two from bacteria chaperones, called HscB and HscA in bacteria. They are highly conserved up to eukaryotes and homologous to DnaJ and DnaK, respectively, but with specific differences. As compared with other chaperones, HscB and HscA have escaped attention and relatively little is known about their functions. After briefly introducing the various chaperone families, we reviewed here the current structural and functional knowledge HscA and HscB and on their role in cluster formation. We critically evaluated the literature and highlighted the weak aspects which will require more attention in the future. We sincerely hope that this study will inspire new interest on this important and interesting system.

## **Abbreviations**

**AD**, Alzheimer disease

**ALS**, amyotrophic lateral sclerosis

**FeS**, iron sulfur

**HSP**, heat shock proteins

**ISC**, iron–sulfur cluster, PD

**PD**, Parkinson's disease

**polyQ**, polyglutamine

The amino acid sequence of a protein is thought to contain all the information required to determine a functional structure in a given environment 1. Nevertheless, in the intracellular environment, the folding of polypeptides could easily undergo misfolding and aggregation triggered by mutations, stress conditions, crowding, confinement, or other causes. Protein folding *in vivo* is thus not always spontaneous and the various organisms have evolved a family of highly conserved proteins called molecular chaperones that have essential roles in many cellular processes, including protein folding, targeting, transport, degradation and signal transduction 2. The function of chaperones is to mediate the correct folding of other proteins by targeting misfolded structures and preventing protein aggregation 3.

In recent years, molecular chaperones have assumed an increasing importance for health in view of the several neurodegenerative disorders characterized by conformational changes in proteins that experience misfolding, aggregation and intra‐ or extraneuronal accumulation as amyloid fibrils. Examples of misfolding diseases are Alzheimer's disease (AD), Parkinson's disease (PD), amyotrophic lateral sclerosis (ALS), and related polyglutamine (polyQ) expansion diseases. Although the protein culprits that aggregate in these disorders are unrelated in size, function, and primary sequence, the resulting aggregates typically contain fibrillar, amyloid‐like structures which are detergent insoluble and protease resistant, with a high β‐sheet content and a cross‐β structure 4. In addition to the ubiquitin–proteasome system and the lysosomes, molecular chaperones provide an important line of defense against misfolded, aggregation‐prone proteins and are among the most potent suppressors of neurodegeneration known in animal models of human diseases 3. Neurons and postmitotic cells cannot dilute potentially toxic species through cell division so that the misfolded proteins accumulate, also as a consequence of a decreased proteasome activity and chaperone function. Toxic species initiate a cascade of pathogenic protein–protein interactions that culminates in neuronal dysfunction and death. Fibrillar aggregates are inert or possibly even protective rather than being directly pathogenic because they can sequester toxic proteins 5. Plaques and inclusion bodies that are characteristic of AD, PD, ALS, and PolyQ diseases colocalize with chaperones and components of the ubiquitin–proteasome degradation system 6. This reflects an irreversible transition toward an aggregated state with consequent loss of function caused by a failed attempt to refold or degrade the aggregated proteins.

The scope of this mini‐review is to summarize the information available on molecular chaperones with specific attention for those involved in iron–sulfur (FeS) cluster biogenesis, an essential pathway which is still relatively underexplored 7, 8, 9. Being aware that the large plethora of excellent review articles on chaperones, we remand the readers to other more complete reviews for a more general perspective on chaperones which may cover complementary aspects 3, 10, 11, 12 and the articles in the recent issue in J. Biol. Inorg. Chem, 2018, Vol 23, Issue 4). We apologize if some important papers have unintentionally been neglected.

## Heat shock proteins

Temperature increase activates the cellular heat shock response with consequent increase in heat shock protein (Hsp) production. Hsps act as chaperones and are essential for the recovery from stress‐induced damage 13. They are classified into six families, Hsp100, Hsp90, Hsp70, Hsp60, Hsp40, and small Hsp (sHsp), according to their molecular weight. They are present in essentially all organisms, prokaryotes, archaea, and eukaryotes, and in almost all compartments. Hsp100s belong to the AAA+ protein family (ATPase with diverse activities) with a typical ring‐shaped structure. Hsp90s reside in various cellular locations such as cytosol, mitochondria, and the endoplasmic reticulum where they stabilize misfolded proteins and regulate the activity of various signaling proteins. Hsp70s assist folding of several substrates in combination with their Hsp40 cochaperones and have a conserved ATPase domain and a C‐terminal substrate domain. Hsp60 are homo‐heptameric complexes with a double‐ring structure that contains a large central cavity in which proteins bind and fold. Eukaryotic members of this family are chaperonines from groups I and II. The Hsp40 family contains cochaperones that bind Hsp70s through a conserved J‐domain. They promote ATP hydrolysis resulting in a conformation change that facilitates recognition of non‐native protein substrates. Hsp40s also bind and chaperone protein substrates to Hsp70s enhancing the efficiency of the refolding cycle. Finally, the sHsp chaperones are small proteins (molecular mass around 40 kDa) that assemble into large oligomeric structures. They transiently interact and stabilize misfolded substrates until the Hsp70‐Hsp40 system can actively refold them 3.

## The Hsp70 family

Hsp70 chaperones are activated by stress response and are involved in folding newly synthesized and misfolded proteins, preventing protein aggregation and cellular trafficking. They also help translocation of proteins across membranes as well as assembly/disassembly of oligomeric structures 14. Both the sequences and the structures of Hsp70s are highly conserved among all species 15. They contain two domains: the nucleotide binding domain (NBD) at the N terminus and the substrate‐binding domain (SBD) at the C terminus. The activity of this family of chaperones is correlated with their ability to bind and hydrolyze ATP to ADP. The general view is that switching between the low‐affinity ATP‐bound and the high‐affinity ADP‐bound conformations results in the release of the substrate. In turn, ATPase activity is stimulated by binding of the substrate or the cochaperone and further enhanced by the presence of both 16, 17. The cochaperone thus controls the ATPase cycle by targeting the Hsp70 protein and the substrate 18. Nonetheless, this hypothesis does not explain old results, whereby Hsp70s with ATP, in the presence of cochaperones, bind substrates better than with ADP. If the ADP state was the “high affinity one” and hydrolysis was just used to switch to the high‐affinity state, we would expect the binding to match the one of the high‐affinity state. As an alternative, it was proposed that this observations could be explained only from a nonequilibrium perspective as suggested by Zuiderweg *et al*. 19 and formally worked out by De Los Rios and Barducci 20.

In prokaryotes, another Hsp70 homolog was identified in addition to the stress‐related Hsp70 DnaK and called HscA. 21. As other Hsp70 proteins, coupled with ATP binding and hydrolysis, HscA has general chaperone‐like activities (prevention of aggregation and protein folding assistance) and acts with the coexpressed the J‐protein cochaperone, HscB, which stimulates the ATPase activity. Despite these similarities, HscA shares only 40% sequence identity with other Hsp70s 22. Also, differently from DnaK, HscA does not require a nucleotide exchange factor, GrpE for maximal activity 23. Its main role is supposed to be related to FeS cluster biogenesis as demonstrated by the inactivation of genes encoding both HscA and HscB which reduces formation of FeS cluster proteins 24, 25. Ssq1/Jac1 and Ssc1/Jac1 are Hsp70 and cochaperones found in specific fungi and in other eukaryotes. Studies of the phylogenetic distribution demonstrated that Ssq1 is more closely related to DnaK than to HscA, suggesting that Ssq1 evolved before HscA. If this were true, this would imply that specialized Hsp70s with a role in FeS cluster biogenesis may have arisen twice in evolution 26. Chaperone systems in bacteria and in mitochondria have significant biochemical differences: Ssq1 binds the mitochondrial nucleotide exchange factor Mge1 and has a higher affinity for ATP than HscA 27, 28, 29.

## The structure of HscA and HscB and their complexes

Dysfunctions in FeS protein biogenesis and mitochondrial iron accumulation in heart and neurons are part of the phenotype of a genetic neurodegenerative disease called Friedreich's ataxia. This pathology is caused by the deficiency of a mitochondrial protein, frataxin, highly conserved throughout species 30 and currently thought to be a regulator of FeS cluster biosynthesis 31, 32. The biogenesis of FeS clusters is an essential process that involves a complex molecular machine with macromolecular structures containing multiple subunits with specific functions. The *isc* operon is one of the three machines responsible for FeS cluster biogenesis in bacteria. It presents a high level of conservation with the homologous eukaryotic system 33. Among the bacterial *isc* components are the desulfurase IscS, the scaffold protein IscU on which the FeS cluster is assembled, an alternative scaffold IscA, the two chaperones HscA and HscB, the transcriptor regulator IscR, ferredoxin (Fdx), and the modulator IscX. The names of these proteins are confusing because they can change completely in the close eukaryotic orthologs.

FeS cluster biosynthesis can be simplified in two steps: first the FeS cluster is assembled on IscU and is then transferred to the target apo‐proteins 34. *In vitro* studies have suggested that the two chaperones may assist FeS cluster formation by maintaining the scaffold protein in a conformation suitable for cluster assembly through interacting with the loaded scaffold protein IscU. Alternatively, it was suggested that HscA may facilitate cluster transfer from cluster‐loaded (holo)‐IscU to other acceptor cluster‐free (apo‐) proteins 35.

**Figure 1:**
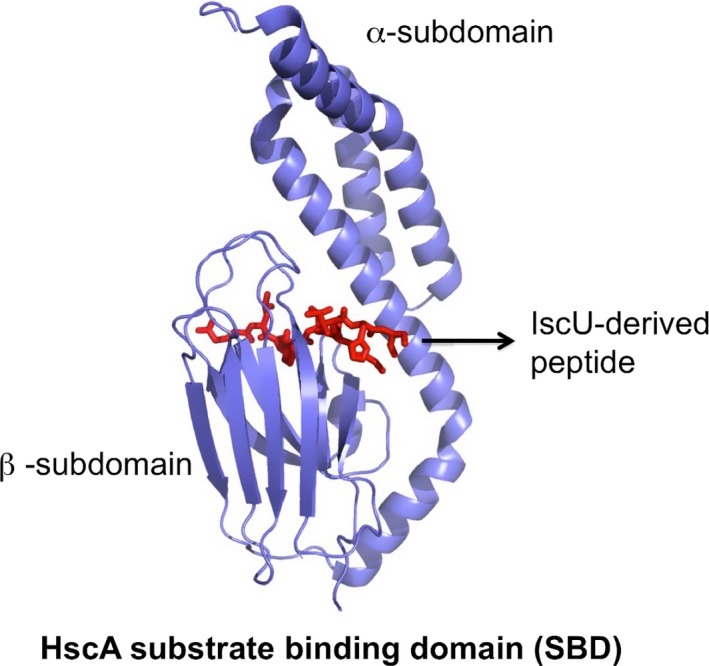
Crystal structure of the complex of the substrate‐binding domain fragment (SBD) of HscA and the IscU‐derived peptide ^98^ ELPPVKIHC ^106^. (PDB: 1U00).

A full understanding of the role of HscA/Ssq1 and HscB/Jac1 requires detailed structural information of the chaperone, cochaperone, and substrates. Several efforts have been spent to determine the structures of HscA and of its complex with the scaffold protein IscU. The scaffold protein interacts with the chaperone through the recognition sequence LPPVK 36, 37 and binding is stabilized by the presence of the cochaperone which enhances ATPase activity 35, 38. A crystal structure of the complex of the substrate‐binding domain fragment (SBD) of HscA and a IscU‐derived peptide (^98^ELPPVKIHC^106^) is available (Fig. 1) (PDB code 1U00 36). This structure has revealed that SBD consists of two distinct subdomains (the α‐ and β‐subdomains) and is overall similar to the SBD of DnaK 39, although the α‐helical subdomain is shifted relative to the β‐subdomain. The IscU peptide binds in an extended conformation in a hydrophobic cleft of the β‐subdomain through a nonpolar and hydrogen bond interactions which contribute to the binding affinity.

**Figure 2:**
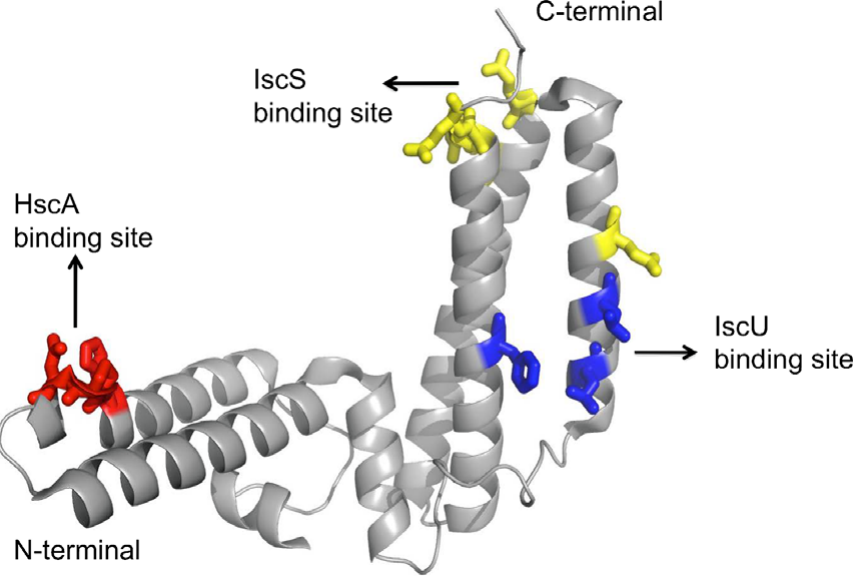
Crystal structure of cochaperone HscB shows two domains: an N‐terminal J‐domain and a C‐terminal domain (PDB: 1FPO). Residues involved in the binding with HscA are colored in red. Residues responsible for the binding with IscU and IscS are in blue and yellow, respectively.

The crystal structure of the isolated cochaperone HscB is also available (PDB code 1FPO 40). It reveals the presence of two distinct domains: an N‐terminal J‐domain and a C‐terminal domain that consists of a three‐helix bundle (Fig. 2). In contrast to DnaJ, HscB does not have intrinsic chaperone activity and lacks the C‐terminal domain necessary for interaction with unfolded polypeptides 35. It has instead a C‐terminal domain that is involved in binding to the substrate, the scaffold protein IscU, through residues L92, L96, and F153 41, 42, 43. These features are not unique and are shared within the subclass of Class C J‐domain chaperones 44.

The J‐domain of HscB is responsible for the interaction with HscA through residues H32, P33, and D34 45. The two domains make contacts with each other through an extensive hydrophobic interface, the presence of which suggests that their relative orientations are fixed. This observation could imply that HscB may function as a scaffold to facilitate positioning of the substrate on HscA in addition to having a role in the regulation of the ATPase activity 40.

More recently, it was described that a previously undetected weak interaction between the HscB and the desulfurase IscS, one of the two main players of the *isc* machine 46. It was shown that the surface of interaction involves a region of HscB in the longer stem of the approximately L‐shaped molecule which binds a cavity of IscS near the active site of the enzyme. Interestingly, this region overlaps with the surface of IscS involved also in binding to ferredoxin, frataxin, and the small prokaryotic‐specific IscX 32, 47, 48.

## The mechanism of the ATPase reaction cycle

The mechanisms involved in IscU binding and release are not well understood. HscA exhibits a low intrinsic ATPase activity (0.6 min^−1^ at 37 °C and pH 7.5) but HscB was found to stimulate this activity up to 3.8‐fold. To better understand the mechanism and regulation of HscA, Silberg and Vickery investigated the kinetics of ATP hydrolysis and proposed a model for the ATPase reaction cycle 49. According to this model, HscA binds ATP by a two‐step process in which HscA is converted from a high peptide affinity (R‐state) to a low peptide affinity (T‐state). The rates of binding and release are faster for the T‐state than for the R‐state complex 38. This implies that ATP hydrolysis is the rate‐limiting step subject to cochaperone regulation 49 and suggests that conformational changes involving the α‐subdomain could be required depending on the nucleotide‐bound state of the chaperone.

**Figure 3:**
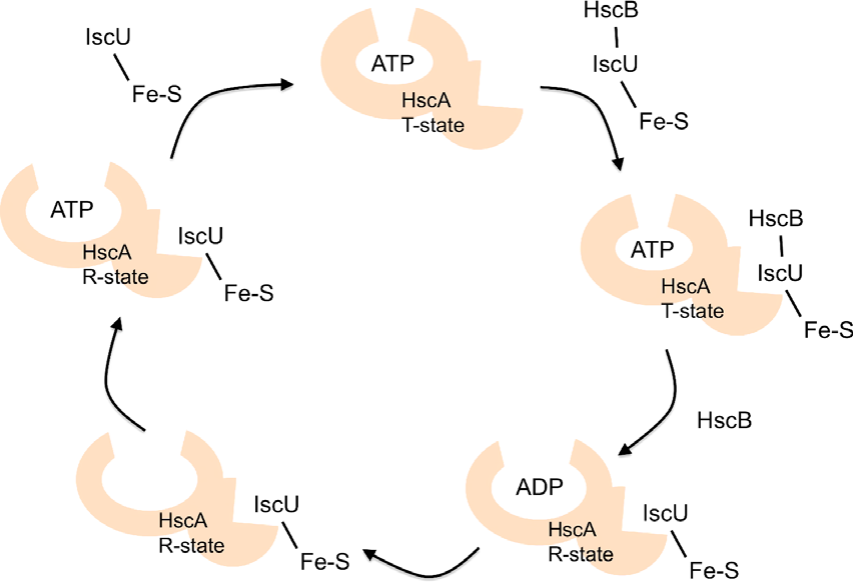
Model of IscU‐binding cycle of HscA. The figure evidences HscA conformational changes associated with substrate‐binding and ATPase activity. The scheme was adapted from 23.

HscA interacts with the scaffold protein IscU and the interaction was shown to stimulate ATPase activity 35. In turn, ATP destabilizes HscA‐IscU complexes allowing their release 50. IscU also interacts with HscB which may serve to control the association of IscU with HscA since, in the presence of HscB, the affinity of IscU for HscA is increased >18‐fold. HscB has also a synergistic effect on IscU stimulation of the ATPase activity of HscA, increasing the rate >50‐fold over that found at saturating levels of IscU alone 35. To better understand the mechanism by which HscB and IscU regulate HscA, Silberg *et al*. examined their binding to the different conformational states of HscA and their effects on the kinetics of the individual steps of the ATPase reaction cycle 38. While IscU binds both ADP (R‐state) and ATP (T‐state)‐HscA complexes, HscB interacts only with an ATP‐bound state. Both IscU and HscB modestly accelerate the rate‐determining step in the HscA reaction cycle, that is, the hydrolysis of ATP. When present together, an enhancement of the HscA(ADP)‐IscU complex formation is observed. Following the ADP/ATP exchange, IscU seems also to stimulate the HscA transition from the R‐ to the T‐state thereby accelerating the rate at which the HscA‐ATP is regenerated 38 (Fig. 3).

## Models of the interaction of the chaperones with IscU and FeS cluster transfer

Chandramouli *et al*. studied cluster transfer from IscU to target apo‐Ferredoxin mediated by HscA and HscB chaperones 51. Since ATP hydrolysis and the presence of HscB accelerate this process, the authors suggested that conformational changes accompanying the ATP (T‐state) to ADP (R‐state) transition in the HscA chaperone could be required for catalysis. ATP binding to HscA would lead to a tense (T) state with decreased substrate‐binding affinity. HscB would bind IscU and escort it to HscA. The presence of HscB would enhance IscU binding to HscA in the ATP‐bound T‐state and lead to a transient HscA‐ATP‐HscB‐IscU complex. This would undergo ATP hydrolysis and loss of HscB to yield an ADP‐bound relaxed (R) state with increased affinity for IscU. The IscU substrate would subsequently be released after ADP/ATP exchange and the R to T transition that occurs upon ATP binding to HscA. As a result, the authors suggested two alternative mechanistic schemes for a chaperone‐catalyzed FeS cluster transfer from FeS‐IscU to apo‐acceptor proteins 51. The first involves direct coupling between ATP hydrolysis and cluster transfer and requires interaction between apo‐Fdx and the T‐state HscA‐ATP‐HscB‐IscU complex. The second involves ATP hydrolysis preceding cluster transfer and interaction between apo‐Fdx and the R‐state HscA‐ADP‐IscU complex. The chaperones could cause changes in IscU conformation that facilitate cluster release or capture by the acceptor protein 52.

**Figure 4:**
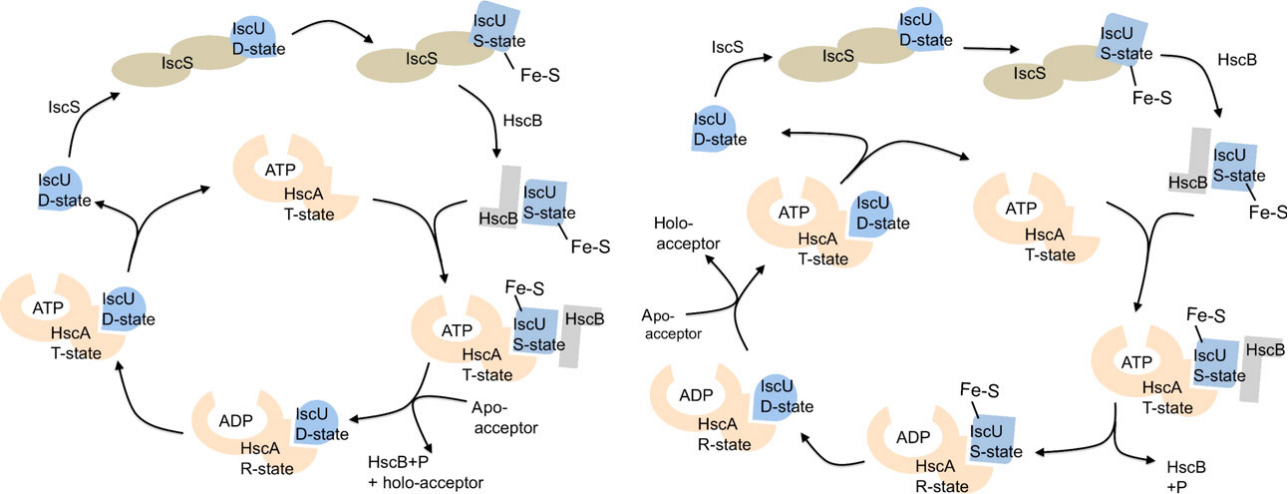
Two possible models of the mechanism of chaperone‐mediated FeS cluster transfer. Proteins involved undergo conformational changes. IscU is supposed to switch from a disordered to a structured form. HscA exists in two conformations with a low and a high affinity for ATP. The figure was adapted from 51. The two models differ by the stage at which an acceptor intervene and the possible placement of the D and S states of IscU.

These two mechanisms are further complicated by the hypothesis suggested by the Markley's group that IscU could exist as an equilibrium between a structured conformation (S) and a partially disordered one (D). If this were true, cluster formation could stabilize the S‐state, which could have a lower affinity for IscS and detach from it 53. At this point, the S‐state would be selectively recognized by HscB 54. The holo IscU‐HscB complex would then represent a target for HscA in its ATP‐bound form. After formation of HscA(ADP), the equilibrium between the IscU conformations would be shifted toward the D‐state ensuring release of the cluster 55. This possibility would lead to two alternative schemes which are the direct consequences of the Chandramouli and Johnson's models (Fig. 4).

## HscA and HscB in FeS cluster biogenesis

Several studies have been performed investigating the effects of the HscA/HscB chaperones on specific steps of the FeS cluster biosynthesis leading to controversial results. Kinetic studies of FeS cluster transfer from holo‐IscU to apo‐Fdx in the presence of *T. maritima* chaperone DnaK‐DnaJ demonstrated an inhibitory effect on the rate of FeS cluster transfer from IscU 56. In contrast, Chandramouli *et al*. observed that the HscA‐HscB complex from *A. vinelandii* facilitates FeS cluster transfer *in vitro* from the holo‐IscU scaffold protein to apo‐Fdx and that cluster transfer is an ATP‐dependent process 51. A study of the chaperones from *E. coli* showed that the HscA‐HscB‐ATP complex, as well as isolated HscB, have an inhibitory effect when the cluster is formed on IscU and independently from the source of sulfur (chemical or enzymatic). In addition, in the presence of only HscB, the cluster assembled on IscU is transferred to apo‐Fdx at a slower rate than in the absence of HscB unless ATP and HscA are also present 52, 57. A more recent investigation showed that HscB, in isolation or in copresence of HscA with or without ATP, could slow down the rate of enzymatic cluster formation independently from the final acceptor 46. This was explained by an interaction between HscB with the desuflurase IscS near the catalytic site which would restrict the motion of the IscS catalytic loop from the catalytic site to IscU to transfer the persulfide 31.

## Conclusions and open questions

Despite considerable progresses in understanding the function of the two chaperones HscA and HscB (and their homologs in eukaryotes) in FeS cluster biogenesis, several aspects remain to be clarified. First of all, it remains an open question why chaperones are needed given that the scaffold protein readily transfers the cluster to other acceptors without the need of any assistance 58. More attention should be paid to find cases in which the cluster transfer could be problematic and would thus require an active role of the chaperones. It is also rather obscure why the cochaperone HscB should bind the desulfurase IscS 46: the significance of this interaction is unclear if we accept tout court the hypothesis that the chaperone function is only to assist the cluster transfer from IscU after its detachment from the enzyme. The importance of ATP in restoring the native state of the substrates according to the De Los Rios and Goloubinoff's model 59 should also be clarified. Full understanding of the molecular bases of the allosteric regulation of the IscU interaction would certainly require the structure of full‐length HscA both in the ADP/ATP forms. Likewise, the *in vivo* existence of a D state of IscU is far from having been demonstrated. IscU is certainly a marginally unstable protein 60, 61 but it gets fully stabilized in the presence of zinc 61, the cluster 53, and IscS 62. Increasing evidence suggests that zinc could be not just an opportunistic partner in the absence of the cluster but an important factor in the regulation of the machine 63, 64, 65. Further effort thus needs to be directed to establish the state of folding of IscU in the cell. Finally, it should be clarified whether HscB is only a cochaperone or has a more active role in the mechanism and how the HscA and HscB act in a concerted way. These open questions set the path for further studies focused on these two specific chaperones which may also, in turn, explain more general properties of this important family of proteins.
